# Impact of Nitroxyl Radicals on Photovoltaic Conversion Properties of Dye-Sensitized Solar Cells

**DOI:** 10.3390/ma17010077

**Published:** 2023-12-23

**Authors:** Ichiro Imae, Ryosuke Akazawa, Yutaka Harima

**Affiliations:** Department of Applied Chemistry, Graduate School of Advanced Science and Engineering, Hiroshima University, 1-4-1 Kagamiyama, Higashi-Hiroshima 739-8527, Hiroshima, Japan

**Keywords:** dye-sensitized solar cells, nitroxyl radicals, redox activities

## Abstract

Nitroxyl radicals, characterized by unique redox properties, have been investigated for their potential influence on the photovoltaic conversion properties of dye-sensitized solar cells (DSSCs). In this study, we investigated the influence of nitroxyl radicals as donor sites in DSSCs. We observed that the redox activity of nitroxyl radicals significantly enhanced the photovoltaic conversion efficiency of DSSCs; this finding can offer new insights into the application of these radicals in solar energy conversion. Furthermore, we found that increasing the proportion of nitroxyl radicals improved the DSSC performance. Through a combination of experimental and analytical approaches, we elucidated the mechanism underlying this enhancement and highlighted the potential for more efficient DSSCs using nitroxyl radicals as key components. These findings provide new avenues for developing advanced DSSCs with improved performances and sustainability.

## 1. Introduction

Since the report on dye-sensitized solar cells (DSSCs) by Grätzel et al. [1], DSSCs have been actively pursued to promote the use of renewable energy to achieve a sustainable society [2]. In DSSCs, dye molecules adsorbed on titanium dioxide (TiO_2_) absorb solar light, causing electrons to be extracted from the dye to the TiO_2_ electrode, allowing for the external generation of electrical power. However, the efficiency of electron injection from the dye to the TiO_2_ electrode can be significantly affected by the aggregation of the dye molecules on the TiO_2_ electrode. This results in interactions among the dye molecules, such as electron transfer, leading to decreased electron injection efficiency and, consequently, the photovoltaic conversion efficiency of DSSC. Common approaches for overcoming this issue include the co-adsorption of dye molecules with bulky aliphatic carboxylic acids, such as chenodeoxycholic acid (CDCA) [3], and introducing bulky substituents into the dye molecules themselves [4]. These methods increase the distance between the dye molecules adsorbed on TiO_2_, alleviating aggregation. Consequently, interactions between the dye molecules are reduced, leading to an improvement in the electron injection efficiency, ultimately enhancing the photovoltaic conversion efficiency of DSSCs. Furthermore, a groundbreaking DSSC was recently developed by Cao, Hagfeldt, and Grätzel, wherein simply pre-adsorbing a hydroxamic acid derivative to control the assembly of the dye improved the molecular packing, leading to an extremely high conversion efficiency reaching 30% [5]. Thus, although research on perovskite solar cells continues to flourish, research on DSSCs is also being actively pursued.

We previously developed a method for the molecular analysis of the aggregation state of dyes by utilizing the nitroxyl radicals present in 2,2,6,6-tetramethylpiperidin-1-oxyl (TEMPO) [6]. The presence of stable nitroxyl radicals in TEMPO allows for electron spin resonance (ESR) spectroscopy. Typically, nitroxyl radicals, owing to their large anisotropies in *g* value and hyperfine coupling constants (hfc) with the nitrogen atom, considerably influence the orientation and motion properties in ESR spectra. In this study, we devised a spin-probe method by applying the characteristics of ESR-active species, that is, the nitroxyl radicals in TEMPO, to analyze the aggregation state of dye molecules in DSSCs. In the course of our previous studies on spin probe methods using dyes containing TEMPO moieties, we observed an increase in the open-circuit voltage and, consequently, an improvement in the photovoltaic conversion efficiency when dye molecules containing TEMPO were utilized in DSSCs. Nishide et al. successfully applied the redox activity of nitroxyl radicals to electrode materials for chargeable and dischargeable batteries by taking advantage of their stable redox properties [7]. Their work promoted us to hypothesize that the nitroxyl radicals in spin-probe molecules would exhibit a similar redox activity and that this property would also affect the DSSC properties. Subsequently, we confirmed the mechanism underlying this phenomenon by designing and synthesizing novel dyes incorporating the TEMPO moiety and investigating their effects on the photovoltaic conversion properties of DSSCs.

## 2. Materials and Methods

### 2.1. Materials

*n*-Hexane, toluene, tetrahydrofuran (THF), chloroform (CHCl_3_), dichloromethane (CH_2_Cl_2_), methanol (MeOH), ethyl acetate, acetone, 2-propanol, and *N*,*N*-dimethylformamide (DMF) were purified via standard methods and used immediately after purification. Tetrabutylammonium perchlorate (TBAP) and *N*-bromosuccinimide (NBS) were purified by recrystallization from ethanol and benzene, respectively, and dried under vacuum. *p*-Toluene sulfonic acid (*p*-TSA, ≥98.0%), 4-bromophenol (99%), and tri(*tert*-butyl)phosphine (P(*t*-Bu)_3_, 98%) were purchased from Sigma-Aldrich and utilized without further purification. Sodium *tert*-butoxide (*t*-BuONa, ≥98.0%), 1-bromohexane (≥98.0%), 4-amino-2,2,6,6-tetramethylpiperidine 1-oxyl (4-aminoTEMPO, ≥98.0%), phosphorus(V) oxychloride (POCl_3_, ≥98.0%), tetrabutylammonium bromide (Bu_4_NBr, ≥98.0%), potassium acetate (KOAc, ≥97.0%), cyanoacetic acid (≥98.0%), iodine (≥99.0%), lithium iodide (LiI, ≥97.0%), 1,2-dimethyl-3-propylimidazolium iodide (DMPrII, ≥98.0%), phenylhydrazine (≥98.0%), and copper(II) acetate (Cu(OAc)_2_, ≥95.0%) were procured from Tokyo Chemical Industry and used without further purification. Potassium carbonate (K_2_CO_3_, ≥99.5%) and piperidine (≥99.0%) were sourced from Nacalai Tesque and employed without further purification. Palladium acetate (Pd(OAc)_2_, ≥97.0%) and ethylene glycol (≥99.5%) were obtained from Wako Pure Chemical Industries, Ltd. (Richmond, VA, USA), and utilized without further purification.

Fluorine-doped tin oxide (FTO) transparent conductive glass was purchased from Nippon Sheet Glass and cut into pieces with a width of 15 mm and length of 25 mm. Subsequently, they were subjected to ultrasonic cleaning by sequentially employing detergent, distilled water, acetone, and 2-propanol. After ultrasonic cleaning, they were air-dried for over 24 h before use.

TiO_2_ paste (PST-18NR) was obtained from JGC Catalysts and Chemicals, Ltd. (Kawasaki, Japan), and used as received.

### 2.2. Characterization

^1^H nuclear magnetic resonance (NMR) and ESR spectra were recorded using a 500-MHz spectrometer (NMR System 500, Varian Inc., Palo Alto, CA, USA) and an ESR spectrometer (JES-RE1X, JEOL, Tokyo, Japan), respectively. Mass spectra were measured using gas chromatography-time-of-flight mass spectrometry (GC-TOFMS; JMS-T100GCV (AccuTOF GCv 4G), JEOL) and electron spray ionization or atmospheric pressure chemical ionization Fourier-transformed mass spectrometry (ESI- or APCI-FTMS; LTQ Orbitrap XL, Thermo Fisher Scientific, Waltham, MA, USA). UV–VIS absorption and emission spectra were recorded on a Shimadzu UV-3150 spectrophotometer and a Hitachi F-4500 fluorescence spectrophotometer, respectively. Cyclic voltammetry (CV) was performed with a potentiostat/galvanostat (HZ-3000, Hokuto Denko, Tokyo, Japan) using a three-electrode system with a spherical Pt working electrode, Pt wire counter electrode, and Ag/Ag^+^ reference electrode; the supporting electrolyte was 0.1 M TBAP in CH_2_Cl_2_. The reference electrode potential was calibrated using ferrocene after each set of measurements. The potentials references to ferrocene were converted to the normal hydrogen electrode (NHE) standard by adding 0.63 V [8].

### 2.3. Fabrication of DSSCs and Photovoltaic Measurements

The TiO_2_ paste was deposited on a FTO substrate via doctor-blading and sintered for 50 min at 450 °C. The 9 μm-thick TiO_2_ electrode (0.5 × 0.5 cm^2^ in a photoactive area) was immersed in 5 mL of a 0.1 mM dye solution of THF for adsorption of the photosensitizer. DSSCs were fabricated with the dye-adsorbed TiO_2_ as the electrode; Pt-coated glass as the counter electrode; and a solution of 0.05 M I_2_, 0.1 M LiI, and 0.6 M DMPrII in acetonitrile as the electrolyte. The photocurrent density (*J*)–voltage (*V*) characteristics were measured using a potentiostat under simulated solar light (AM 1.5, 100 mW cm^−2^) supplied by a solar simulator (HAL-302, Asahi Spectra, Tokyo, Japan). Incident photon-to-current conversion efficiency (IPCE) spectra were measured under monochromatic irradiation using a tungsten-halogen lamp (AT-100HG, Shimadzu, Kyoto, Japan) and a monochromator (Shimadzu SPG-120 S). We calculated the standard deviation of the conversion efficiencies of multiple DSSCs fabricated with each dye, and we confirmed that the standard deviations were all approximately 0.1.

### 2.4. Synthesis

#### 2.4.1. Compound **1**: 1-Bromo-4-hexyloxybenzene

The synthesis of 1-bromo-4-hexyloxybenzene was performed referencing a previously reported method [9]. Briefly, 4-bromophenol (3.01 g, 17.4 mmol), K_2_CO_3_ (12.05 g, 87.2 mmol), 1-bromohexane (3.17 mL, 22.6 mmol), and acetone (150 mL) were added to a two-neck round-bottom flask. Then, the mixture was stirred at 80 °C under an N_2_ atmosphere for 7 h with reflux. The resulting reaction solution was cooled to room temperature, which precipitated a white solid that was then filtered. The filtered solution was passed through Celite, and the filtrate was concentrated using a rotary evaporator. The obtained pale-yellow liquid was purified using silica gel column chromatography (eluent: dichloromethane/hexane = 4:1) to yield compound **1** with a pale-yellow color. Yield: 3.58 g (13.9 mmol), 79.9%.

^1^H NMR (400 MHz, CDCl_3_, *δ*, ppm): 0.90 (t, *J* = 7.04 Hz, 3H, C***H***_3_), 1.3–1.4 (m, 4H, (C***H***_2_)_2_CH_3_), 1.44 (tt, *J* = 6.70, 6.98 Hz, 2H, O(CH_2_)_2_C***H***_2_), 1.76 (tt, *J* = 6.58, 6.70 Hz, 2H, OCH_2_C***H***_2_), 3.91 (t, *J* = 6.58 Hz, 2H, OC***H***_2_), 6.77 (d, *J* = 9.02 Hz, 2H, phenylene-***H***), and 7.36 (d, *J* = 9.02 Hz, 2H, phenylene-***H***).

#### 2.4.2. Compound **2**: 4-(4-Hexyloxy-phenylamino)-2,2,6,6-tetramethyl-piperidine 1-oxyl

A two-neck round-bottom flask was purged with N_2_. Pd(OAc)_2_ (48 mg, 0.21 mmol), 4-aminoTEMPO (1.55 g, 9.07 mmol), and *t*-BuONa (561 mg, 5.84 mmol) were placed in the flask, adding distilled toluene (80 mL), compound **1** (1.00 g, 3.90 mmol), and P(*t*-Bu)_3_ (0.60 mL, 2.47 mmol); the mixture was stirred at reflux (110 °C) for 9 h. After the reaction, the solution was cooled to room temperature, and chloroform and a saturated saline solution were added to extract the organic layer. The organic layer was dried over anhydrous sodium sulfate (Na_2_SO_4_) and concentrated using a rotary evaporator, resulting in a reddish-brown liquid. The crude product was purified using silica gel column chromatography (eluent: dichloromethane/ethyl acetate = 10:1) to yield compound **2** as an orange-red solid. Yield: 678 mg (1.95 mmol), 50.2%.

High-resolution mass spectrometry (HRMS) (ESI) *m*/*z* calculated for C_21_H_36_O_2_N_2_ = 348.27713 ([M + 1]^+^), found = 348.27731 ([M + 1]^+^).

#### 2.4.3. Compound **3**: 5-Formyl-2,2′-bithiophene

The synthesis of 5-formyl-2,2′-bithiophene was performed using the Vilsmeier–Haack reaction [10]. Briefly, a three-neck round-bottom flask equipped with a dropping funnel and condenser was heat-dried and purged with N_2_. Then, 2,2′-bithiophene (9.19 g, 55.3 mmol) was introduced into this reaction vessel, following which DMF (150 mL) was added under stirring. Another two-necked, round-bottom flask was heat-dried and purged with N_2_; DMF (30 mL) was added to the flask, following which it was placed in an ice bath and POCl_3_ (5.66 mL, 62.0 mmol) was slowly added under stirring. The resulting solution was transferred to a dropping funnel and slowly added dropwise to the 2,2′-bithiophene solution in an ice bath. The mixture was then gradually brought back to room temperature and stirred for 12 h at 80 °C. Subsequently, the reaction mixture cooled to room temperature was poured into water (1.5 L), stirred, and kept overnight. The obtained solution was dissolved in dichloromethane and washed with aqueous sodium acetate and saturated saline solutions. After drying the organic layer with anhydrous Na_2_SO_4_, it was filtered, and the solvent was removed using a rotary evaporator. The resulting dark-green crude product was purified using silica gel column chromatography (eluent: dichloromethane/hexane = 1:1) to yield compound **3** as a light-yellow solid. Yield: 7.87 g (40.5 mmol), 73.2%.

^1^H NMR (400 MHz, acetone-*d*_6_, *δ*, ppm): 7.17 (dd, *J* = 3.67, 5.09 Hz, 1H, 4′-thienyl-***H***), 7.46 (d, *J* = 3.91 Hz, 1H, 3-thienylene-***H***), 7.54 (dd, *J* = 1.08, 3.67 Hz, 1H, 3′-thienyl-***H***), 7.62 (dd, *J* = 1.08, 5.09 Hz, 1H, 5′-thienyl-***H***), 7.92 (d, *J* = 3.91 Hz, 1H, 4-thienylene-***H***), and 9.92 (s, 1H, –C***H***O).

#### 2.4.4. Compound **4**: 5-Formyl-5′-(4-bromophenyl)-2,2′-bithiophene

A round-bottom flask was purged with N_2_ and 1,4-dibromobenzene (1.95 g, 8.27 mmol), compound **3** (411 mg, 2.12 mmol), Pd(OAc)_2_ (23 mg, 0.103 mmol), Bu_4_NBr (669 mg, 2.08 mmol), KOAc (493 mg, 5.02 mmol), and distilled DMF (20 mL) were added. The solution was stirred at 90 °C under an N_2_ atmosphere for 12 h. After completion of the reaction, the solution was cooled to room temperature, washed with dichloromethane and a saturated saline solution, and the organic layer was extracted. The obtained solution was dried over anhydrous Na_2_SO_4_, filtered, and the solvent was removed using a rotary evaporator, resulting in a brown solid. The crude product was purified using silica gel column chromatography (eluent: dichloromethane/hexane = 2:1), yielding compound **4** as a yellow solid. Yield: 230 mg (0.659 mmol), 31.1%.

^1^H NMR (400 MHz, acetone-d_6_, *δ*, ppm): 7.52 (d, *J* = 4.01 Hz, 1H, thienylene-***H***), 7.57 (s, 2H, thienylene-***H***), 7.64 (d, *J* = 8.61 Hz, 2H, phenylene-***H***), 7.69 (d, *J* = 8.61 Hz, 2H, phenylene-***H***), 7.95 (d, *J* = 3.86 Hz, 1H, thienylene-***H***), and 9.94 (s, 1H, –C***H***O).

HRMS (DI) *m*/*z* calculated for C_15_H_9_BrOS_2_ = 347.92782 (M^+^), found 347.92741 (M^+^).

#### 2.4.5. Compound **5**: 2-[5′-(4-Bromophenyl)-2,2′-bithiophen-5-Yl]-1,3-dioxolane

A two-neck round-bottom flask was rigorously purged with N_2_. Compound **4** (155 mg, 0.356 mmol), ethylene glycol (0.98 mL, 17.8 mmol), *p*-TSA (34 mg, 0.178 mmol), and distilled toluene (10 mL) were added to the flask. The solution was refluxed at 110 °C for 24 h. After the reaction, the solution was cooled to room temperature, washed with dichloromethane and a saturated sodium bicarbonate solution, and the organic layer was extracted. The obtained solution was dried over anhydrous Na_2_SO_4_ and filtered, and the solvent was evaporated using a rotary evaporator to yield compound **5** as a pale-yellow solid. The obtained solid was used without further purification to synthesize compound **6**.

^1^H NMR (400 MHz, acetone-*d*_6_, δ, ppm): 7.52 (d, *J* = 4.01 Hz, 1H, thienylene-***H***), 7.57 (s, 2H, thienylene-***H***), 3.96–4.14 (m, 4H, OC***H***_2_C***H***_2_O), 6.04 (s, 1H, C***H***O_2_), 7.16 (d with fine coupling, *J* = 3.71 Hz, 1H, thienylene-***H***), 7.22 (d, *J* = 3.71 Hz, 1H, thienylene-***H***), 7.30 (d, *J* = 3.82 Hz, 1H, thienylene-***H***), 7.48 (d, *J* = 3.82 Hz, 1H, thienylene-***H***), 7.61 (d, *J* = 9.03 Hz, 2H, phenylene-***H***), and 7.64 (d, *J* = 9.03 Hz, 2H, phenylene-***H***).

#### 2.4.6. Compound **6**: 4-[N-(4-(5′-(1,3-Dioxolan-2-Yl)-2,2′-bithiophen-5-Yl)phenyl)-N-(4-hexyloxy-phenyl)amino]-tetramethylpiperidine 1-oxyl

A two-neck round-bottom flask was purged with N_2_, and **2** (290 mg, 0.834 mmol), **5** (298 mg, 0.758 mmol), Pd(OAc)_2_ (11 mg, 0.049 mmol), *t*-BuONa (111 mg, 1.16 mmol), distilled toluene (15 mL), and P(*t*-Bu)_3_ (0.50 mL, 2.06 mmol) were added. The mixture was refluxed at 110 °C for 12 h under stirring. After cooling to room temperature, the solution was washed with dichloromethane and saturated saline solution. The organic layer was dried over anhydrous Na_2_SO_4_ and filtered, and the solvent was removed using a rotary evaporator. The resulting oily red substance was vacuum dried. The crude product was purified using silica gel column chromatography (eluent: dichloromethane) to yield compound **6** as a yellow-brown solid. Yield: 212 mg (0.322 mmol), 39.1%.

HRMS (DI) *m*/*z* calculated for C_38_H_47_N_2_O_4_S_2_ = 659.29772 (M^+^), found = 659.29630 (M^+^).

#### 2.4.7. Compound **7**: 4-[N-(4-(5′-Formyl-2,2′-bithiophen-5-Yl)phenyl)-N-(4-hexyloxy-phenyl)amino]-tetramethylpiperidine 1-oxyl

Compound **6** (212 mg, 0.322 mmol), *p*-TSA (31 mg, 0.161 mmol), distilled water (6.5 mL), and distilled THF (20 mL) were added to a two-necked round-bottom flask, and the mixture was stirred at room temperature for 24 h. After removing THF using a rotary evaporator, dichloromethane and a saturated sodium bicarbonate solution were added, and the organic layer was extracted. The organic layer was dried over anhydrous Na_2_SO_4_ and filtered, following which the solvent was removed using a rotary evaporator, resulting in an orange solid. As no byproducts were observed using thin-layer chromatography, they were used without purification in the synthesis of the next compound. Yield (crude): 130 mg.

The HRMS (APCI) *m*/*z* calculated for C_36_H_44_O_3_N_2_S_2_ = 616.27879 ([M + 1]^+^), found 616.27863 ([M + 1]^+^).

#### 2.4.8. Compound **8**: 2-Cyano-3-(5′-{4-[N-(4-hexyloxy-phenyl)-N-(1-oxyl-2,2,6,6-tetramethyl-piperidin-4-Yl)amino]phenyl}-2,2′-bithiophen-5-Yl)acrylic Acid (**TEMPO-Dye**)

The compound **7** (130 mg, 0.211 mmol), cyanoacetic acid (54 mg, 0.633 mmol), piperidine (180 mg, 2.11 mmol), and CHCl_3_ (10 mL) were added to a two-neck round-bottom flask, and the mixture was stirred at 70 °C under an N_2_-atmosphere for 12 h. After the reaction was completed, the solution was cooled to room temperature, hydrochloric acid (pH = 3) was added, and the organic layers were extracted. The organic layer was dried over anhydrous Na_2_SO_4_, filtered, and the solvent was removed using a rotary evaporator, resulting in a purple solid. The crude product was purified by silica gel column chromatography (eluent: dichloromethane/methanol = 10:1), yielding **TEMPO-dye** as a dark red solid. Yield: 53.3 mg (0.078 mmol), 37.0%.

HRMS (APCI) *m*/*z* calculated for C_39_H_44_O_4_N_3_S_2_ = 682.27677 (M^+^), found = 682.27754 (M^+^).

## 3. Results

### 3.1. Design and Synthesis of Dye Compound

We adopted a donor–π–acceptor (D–π–A) structure for the molecular framework of the dye, incorporating an aromatic amine as the donor moiety, bithiophene as the π-linker, and cyanoacrylic acid as the acceptor moiety. Generally, in this molecular framework, the highest occupied molecular orbital (HOMO) was localized on the donor moiety and the lowest unoccupied molecular orbital (LUMO) was localized on the acceptor moiety. During light irradiation, electrons from the donor moiety underwent intramolecular charge transfer (ICT) to the acceptor moiety via the π linker. Furthermore, the carboxylic group on the acceptor moiety adsorbed onto the surface of the TiO_2_ electrode, facilitating the smooth injection of the transferred electrons into the TiO_2_ electrode. In our previous study, we also employed the D–π–A framework for the dye incorporating TEMPO as a spin probe. However, highly reactive N–H groups were retained in the donor moiety in these spin probe dyes. Therefore, to prevent unexpected side reactions that might have occurred with these N–H groups, a phenyl group was introduced into the donor moiety in the dye molecule in the present study.

**Scheme 1:**
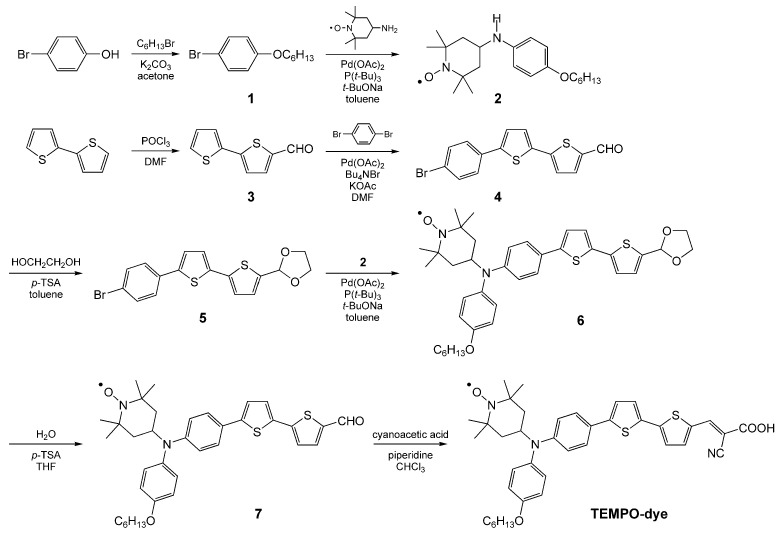
Synthesis of **TEMPO-dye**.

The dye was synthesized as shown in Scheme 1. The synthesis of the donor moiety involved a Buchwald–Hartwig reaction [11] between **1** and 4-aminoTEMPO. The π-linker moiety, compound **4**, was synthesized through a direct C–H arylation reaction [12] between 5-formyl-2,2′-bithiophene (compound **3**) and 1,4-dibromobenzene. The coupling of the donor and π-linker moieties was achieved by protecting the formyl group of compound **4** as an acetal [13], followed by a Buchwald–Hartwig reaction. After deprotection of the protecting group, the final target, a D–π–A type dye referred to as **TEMPO-dye**, was synthesized through the Knoevenagel condensation [14] of compound **7** with cyanoacetic acid. Owing to the presence of paramagnetic radical species in the compounds containing the TEMPO moiety (compounds **2**, **6**, **7**, and **TEMPO-dye**), NMR identification was challenging because the strong interaction between electronic spins and hydrogen nuclear spins caused broadening of the signals. However, accurate molecular weights were confirmed by HRMS (see also Figure S1 in Supplementary Materials), and the results indicated the successful synthesis of the target compounds. For **TEMPO-dye**, an additional signal corresponding to a hydrogen adduct formed by the deactivation of the radical species (calculated for C_39_H_46_O_4_N_3_S_2_ = 684.29242 ([M + H]^+^), found = 684.29304 ([M + H]^+^)) was also observed, in addition to a signal corresponding to the molecular weight of the target compound (calculated for C_39_H_44_O_4_N_3_S_2_ = 682.27677 (M^+^), found = 682.27754 (M^+^)). In the positive mode of HRMS, the molecular ion peak of TEMPO-dye was observed, and its theoretical value agreed well with the calculated value up to two decimal places, confirming the correctness of its molecular formula. However, the signal intensity was weak and obscured by noise. In contrast, in the negative mode of HRMS, the ion peak of the target compound could be clearly identified.

### 3.2. Calculating Proportion of Residual Radicals and Controlling It via Reaction Time

**Figure 1:**
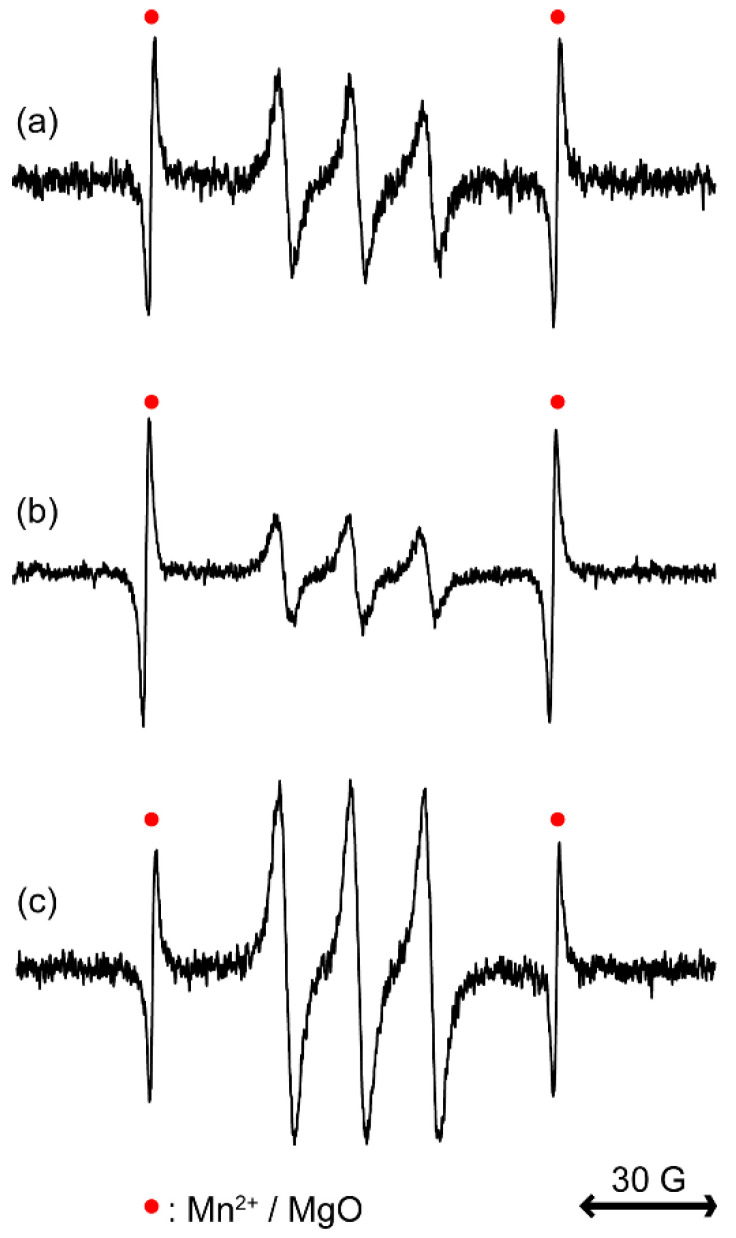
ESR spectra of (**a**) *pristine-* (**b**) *red-*, and (**c**) *ox-***TEMPO-dye**s.

As mentioned earlier, the nitroxyl radicals of freshly synthesized **TEMPO-dye** (hereinafter referred to as *pristine-***TEMPO-dye**) were partially deactivated, resulting in the generation of hydrogen-addition products. To calculate the proportion of residual nitroxyl radicals in the obtained product, ESR spectra were measured. Figure 1a illustrates the ESR spectrum of *pristine-***TEMPO-dye**, which exhibits the characteristic equidistant triplet lines associated with nitroxyl radicals. The proportion of residual radicals in **TEMPO-dye** molecules (*C*_radical_) was estimated using the following equations:

*C*_radical_ = *x*/*y*, (1)

*x* = (*S*(**TEMPO-dye**)/(*S*(M_n_)), (2)

*y* = (*S*(TEMPO))/(*S*(M_n_)), (3)

where *S*(**TEMPO-dye**), *S*(TEMPO), and *S*(Mn) correspond to the double integrated values of **TEMPO-dye**, TEMPO with a clear spin concentration (99%), and the manganese marker used in ESR spectra, respectively. It was calculated that the proportion of the residual radicals in *pristine-***TEMPO-dye** was 43%.

**Figure 2:**
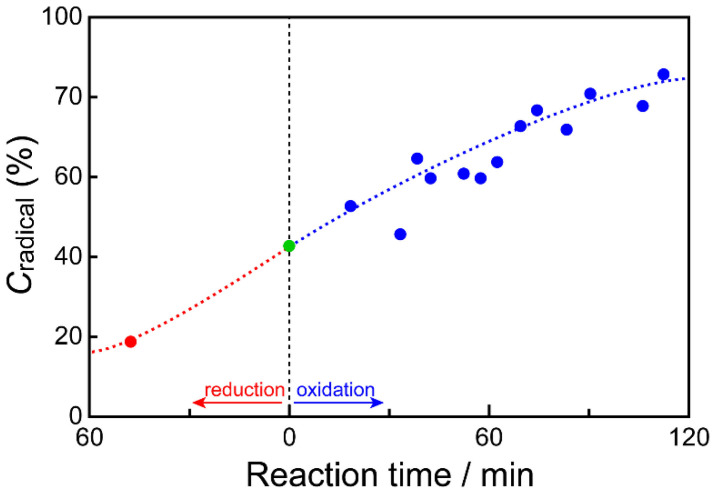
Modification of the proportion of residual nitroxyl radicals in **TEMPO-dye**.

To modify the proportion of residual radicals in **TEMPO-dye**, we treated samples with phenylhydrazine as a reducing agent and copper acetate as an oxidizing agent; the resulting dyes are hereinafter referred to as *red-***TEMPO-dye** and *ox-***TEMPO-dye**, respectively. The ESR signal of *red-***TEMPO-dye** exhibited a reduced intensity, whereas that of *ox-***TEMPO-dye** exhibited an increased intensity. These results indicate that in *red*-**TEMPO-dye**, the proportion of residual radicals decreased as the nitroxyl radicals were reduced to N–OH groups by phenylhydrazine, while in *ox*-**TEMPO-dye** the proportion increased as the N–OH groups were oxidized back to nitroxyl radicals by copper acetate. The change in the proportion of the residual radicals could be controlled by varying the reaction time with each reagent. Therefore, the proportion of the residual radicals in **TEMPO-dye** was successfully varied between 19% and 86% (Figure 2). In this experiment, it was not possible to synthesize dye molecules with 0% or 100% residual radicals. However, given that this study aimed to investigate the influence of this proportion on DSSC characteristics, it would be ideal to synthesize dye molecules with residual radical proportions of 0% and 100% to more clearly observe these effects. Therefore, in future investigations, we would like to explore DSSC characteristics using dyes with a broader range of proportions, including 0% and 100%. The chemical stability of the dye developed in this study and the durability of the DSSC using it are crucial factors for practical application. However, as this paper focused on preliminary results regarding the unique characteristics of the dye with TEMPO radical, discussions on these factors were insufficient. In the future, we aim to discuss more deeply into these aspects.

### 3.3. Optical and Electrochemical Properties of TEMPO-Dye

**Figure 3:**
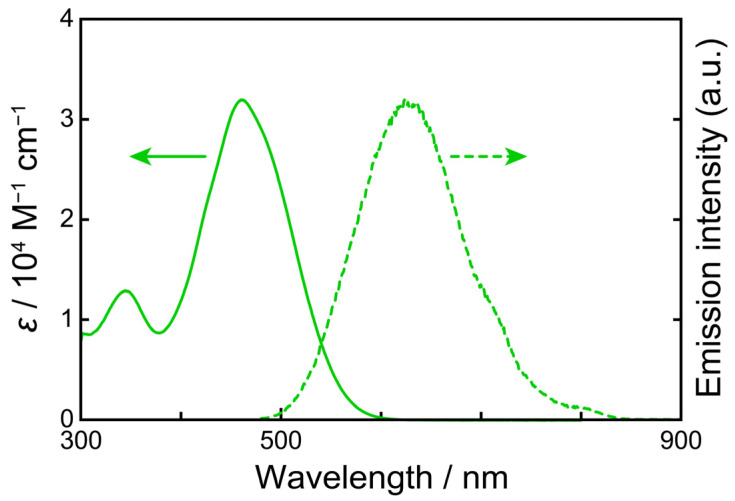
Absorption and emission spectra of **TEMPO-dye**.

The absorption and emission spectra of *pristine-***TEMPO-dye** are presented in Figure 3 and their data are summarized in Table 1. The absorption originating from ICT was observed at approximately 460 nm, while emission was observed at approximately 620 nm. The bandgap of the dye, which was calculated from the intersection of these signals and is known as the zero–zero transition (*E*_0–0_), was determined to be 2.30 eV. The absorption and emission spectra of dyes with different proportions of residual radicals showed no significant differences in the absorption or emission wavelength regions, indicating that the proportion of residual radicals did not significantly affect the optical properties.

**Figure 4:**
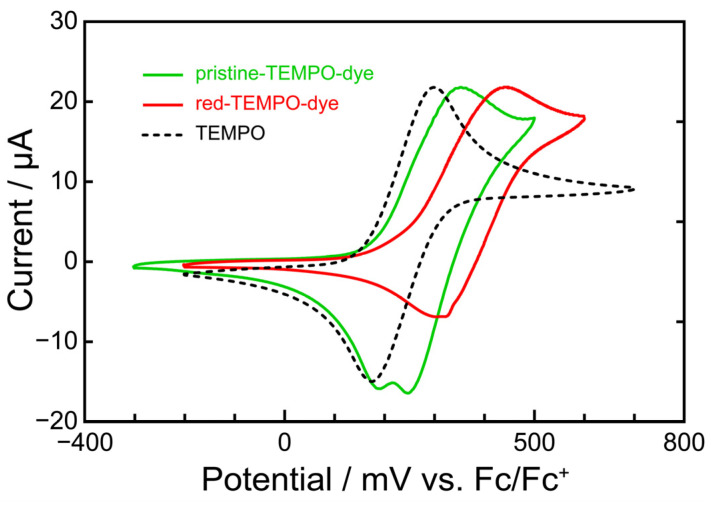
CV curves of *pristine-* and *red-***TEMPO-dye**s and TEMPO.

Figure 4 shows CVs of **TEMPO-dye**s and their data are summarized in Table 1. The HOMO level of the dye was more positive than the redox level of the electrolyte (I_3_^−^/I^−^) (0.4 V vs. NHE), and the LUMO level of the dye was more negative than the conduction band of TiO_2_ (−0.5 V vs. NHE) [15]. Therefore, it is suggested that the dye functioned as a sensitizer for DSSCs. Interestingly, compared with the CV curve of *pristine-***TEMPO-dye**, the CV curve of *red-***TEMPO-dye** was positively shifted. Additionally, while two reduction peaks were observed in the CV curve of *pristine-***TEMPO-dye**, only one reduction peak was observed for *red-***TEMPO-dye**. The high contribution of the overlapping of the oxidation–reduction waves of the TEMPO moiety in *pristine-***TEMPO-dye** caused the reduction wave to shift to the negative side, and each peak could be observed for the reduction wave. In *red-***TEMPO-dye**, however, the lower proportion of residual radicals, which were the redox centers, resulted in a lower contribution of the oxidation–reduction waves of the TEMPO moiety, leading to the observed differences.

### 3.4. Device Characteristics

**Figure 5:**
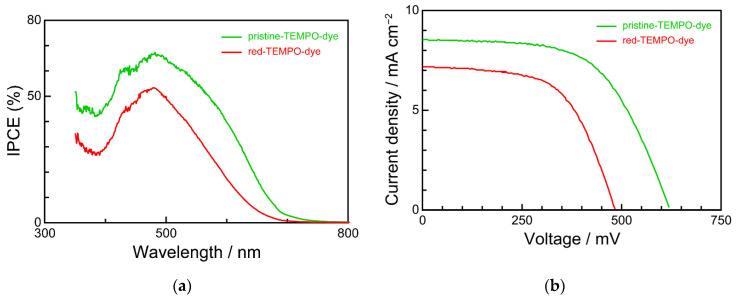
(**a**) IPCE spectra and (**b**) *J*–*V* curves of DSSCs using *pristine-* and *red-***TEMPO dye**s.

We fabricated a DSSC using TiO_2_ coated with **TEMPO-dye** and evaluated its conversion efficiency. When varying the concentration of the dye solution used to adsorb the dye onto TiO_2_, we evaluated the surface coverage of dyes adsorbed on TiO_2_. We found that the surface coverage of adsorbed dyes became constant at a concentration of 0.1 mM and above, indicating that the surface coverage of dyes adsorbed at saturation was 1.4 × 10^14^ [cm^−2^]. This value was comparable to that of dye adsorbed at saturation (1.1–1.7 × 10^14^ [cm^−2^]) previously investigated by our group as a spin-probe dye, confirming that the molecular structure of the dye did not significantly influence the surface coverage of the adsorbed dyes. Therefore, when fabricating DSSCs in this study, the concentration of the **TEMPO-dye** solution for adsorption onto the TiO_2_ electrode was set to 0.1 mM. Figure 5 depicts the IPCE spectrum and characteristic *J*–*V* curves. The IPCE spectrum reflected the optical absorption characteristics of the **TEMPO-dye**, which demonstrated a broad response in the visible light range. Comparing the IPCE characteristics of the devices using *pristine-***TEMPO-dye** and *red-***TEMPO-dye**, we observed a reduction in the maximum value at approximately 500 nm for *red-***TEMPO-dye** to approximately 50% compared with the approximately 65% for *pristine-***TEMPO-dye**. This reduction suggests that the difference in the proportion of residual radicals significantly affected the charge injection efficiency.

We compared the open-circuit voltages (*V*_oc_) and short-circuit current densities (*J*_sc_) obtained from the average values of five devices (Table 2). The short-circuit current density, which reflected the difference in IPCE, was 1.2 times higher for devices using *pristine-***TEMPO-dye** than for those using *red-***TEMPO-dye**. Furthermore, the open-circuit voltages of the devices using *pristine-***TEMPO-dye** and *red-***TEMPO-dye** were 618 and 485 mV, respectively, a difference of 133 mV. Although *J*_sc_ and *V*_oc_ reflected noticeable differences in the proportion of residual radicals, the fill factor (*ff*) of the devices was the same. These differences indicate that the DSSC using the *pristine-***TEMPO-dye** exhibited an approximately 1.5 times higher photoelectric conversion efficiency (*η*) than the DSSC using *red-***TEMPO dye**, indicating that the redox activity of the TEMPO moiety was beneficial to DSSC performance.

In general, DSSCs using D–π–A-type dyes undergo ICT as the dye molecules are excited by light, and electrons from the donor moiety move via the π linker to the acceptor moiety. Several such dyes contain functional groups that, in addition to facilitating dye absorption, allow the acceptor moiety to approach the TiO_2_ electrode. Thus, electrons moving towards the acceptor moiety and approaching the TiO_2_ electrode can be injected through the adsorption site. When the electrons move from the dye molecules to the TiO_2_ electrode, a positive charge remains on the dye molecules. However, if reverse electron transfer from the TiO_2_ electrode to oxidized dye molecules occurs, the open-circuit voltage decreases.

**TEMPO-dye** incorporates the redox-active species TEMPO at the donor site, which has a lower oxidation–reduction potential than aromatic amines. Therefore, when the TEMPO site was active, the positive charge generated after electron injection from the dye to the TiO_2_ electrode moved from the aromatic amine site to the TEMPO site. This stabilization of the positive charge may have suppressed reverse electron transfer from the TiO_2_ electrode, potentially explaining the improvement in the open-circuit voltage of devices when **TEMPO-dye** with a higher proportion of residual radicals was used. Therefore, we concluded that using *ox-***TEMPO-dye**, which has a higher proportion of residual radicals than *pristine-***TEMPO-dye**, can further enhance the photoelectric conversion efficiency of DSSCs. However, owing to the limited sample quantity at present, it is challenging to fabricate devices using *ox-***TEMPO-dye**. We plan to increase the sample quantity in future studies and to investigate the photoelectric conversion properties of devices using *ox-***TEMPO-dye**.

## 4. Conclusions

We developed a novel D–π–A-type dye by incorporating the redox-active species TEMPO at the donor site. The proportion of residual nitroxyl radicals in the TEMPO unit of this dye could be widely controlled using phenylhydrazine and copper(II) acetate. Although the proportion of residual radicals did not affect the light absorption range or bandgap, it significantly influenced the redox properties, indicating a substantial impact on the redox activity of the TEMPO unit. The DSSC fabricated using the TEMPO dye with a higher proportion of residual radicals exhibited a higher IPCE, resulting in a 1.2-fold improvement in short-circuit current values. Moreover, the presence of a redox-active TEMPO unit at the donor site, which possibly suppressed reverse electron transfer, led to a 1.3-fold increase in the open-circuit voltage. These results provide valuable insights into the beneficial effects of suppressing reverse electron transfer in DSSCs and provides new molecular design guidelines for future dye development.

## Figures and Tables

**Table 1:** Optical and electrochemical properties of **TEMPO-dye**.

*λ*_max_ of Absorption /nm	*λ*_max_ of Emission /nm	Band Gap (*E*_0–0_) /V	HOMO/V vs. NHE	LUMO/V vs. NHE
461	624	2.30	0.93	–1.37

**Table 2:** Photovoltaic data of *pristine-* and *red-***TEMPO-dye**s ^(a)^.

Dye	*J*_sc_/mA cm^−2^	*V*_oc_/mV	*ff*/-	*η* (%)
*pristine-* **TEMPO-dye**	8.69	618	0.59	3.16
*red-* **TEMPO-dye**	7.22	485	0.59	2.06

^(a)^ Average values of the data obtained from the five devices.

## Data Availability

The data presented in this study are available in the article.

## Supplementary Materials

The following supporting information can be downloaded at: https://www.mdpi.com/article/10.3390/ma17010077/s1, Figure S1: HRMS spectra of (a) compound **2**, (b) compound **4**, (c) compound **6**, (d) compound **7**, and (e) **TEMPO-dye**.
