# Supplementary material for: Impact of Nitroxyl Radicals on Photovoltaic Conversion Properties of Dye-Sensitized Solar Cells
Source: Materials (Basel). 2023 Dec 23;17(1):77. doi: 10.3390/ma17010077 (PMC10779495; doi:10.3390/ma17010077)
Supplement: Supplementary file 1 [file materials-17-00077-s001.zip › materials-2774008-supplementary.pdf]

Supporting Information

Impact of Nitroxyl Radicals on Photovoltaic Conversion Properties of Dye-Sensitized Solar Cells

Ichiro Imae *, Ryosuke Akazawa, and Yutaka Harima

(a)

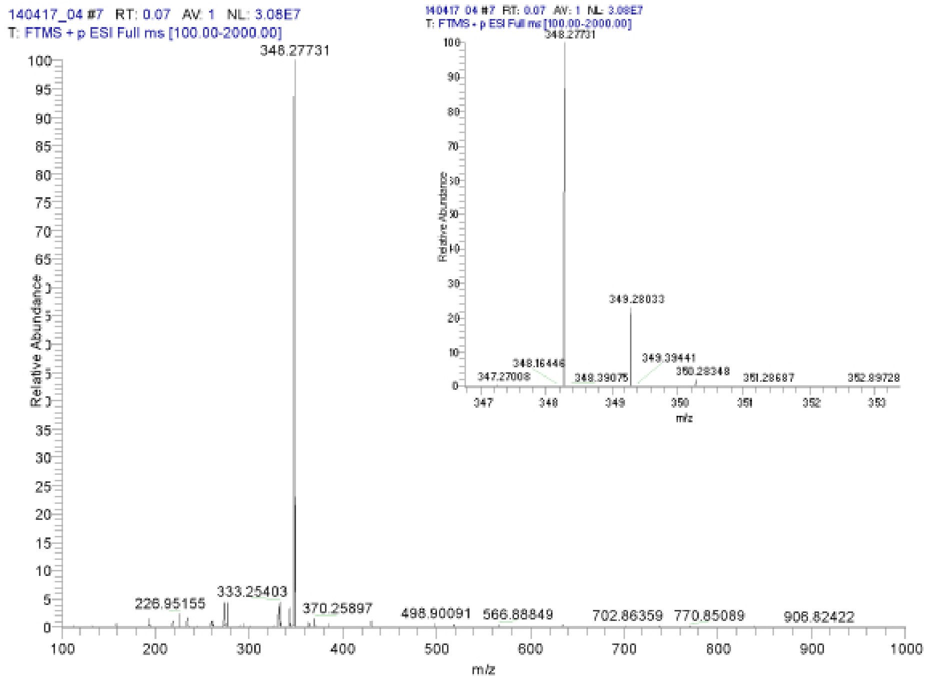

(b)

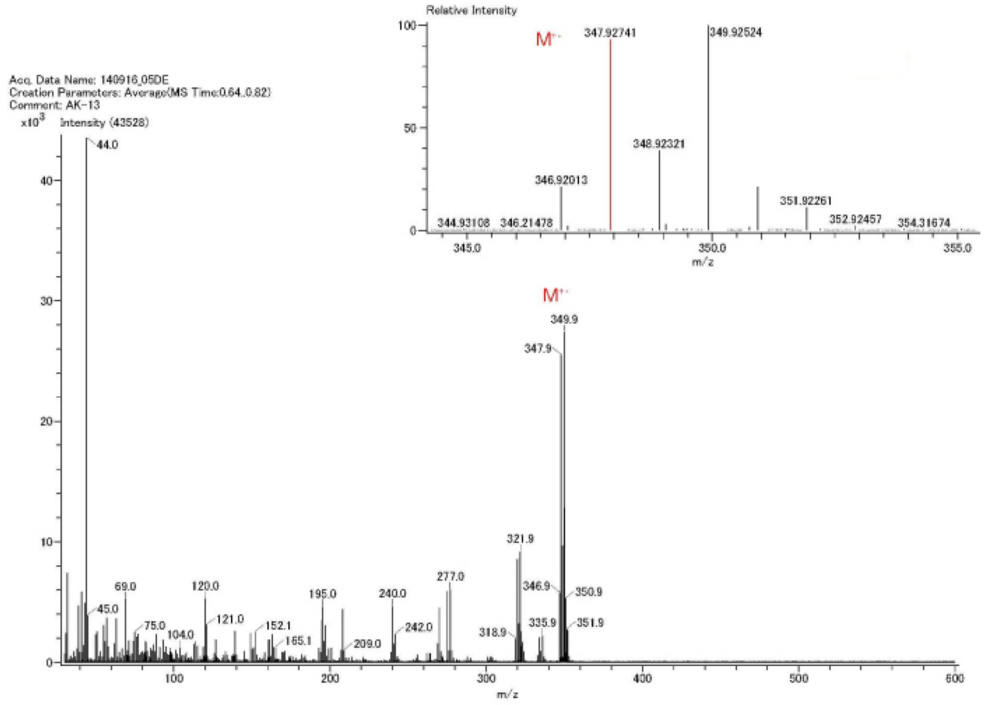

(c)

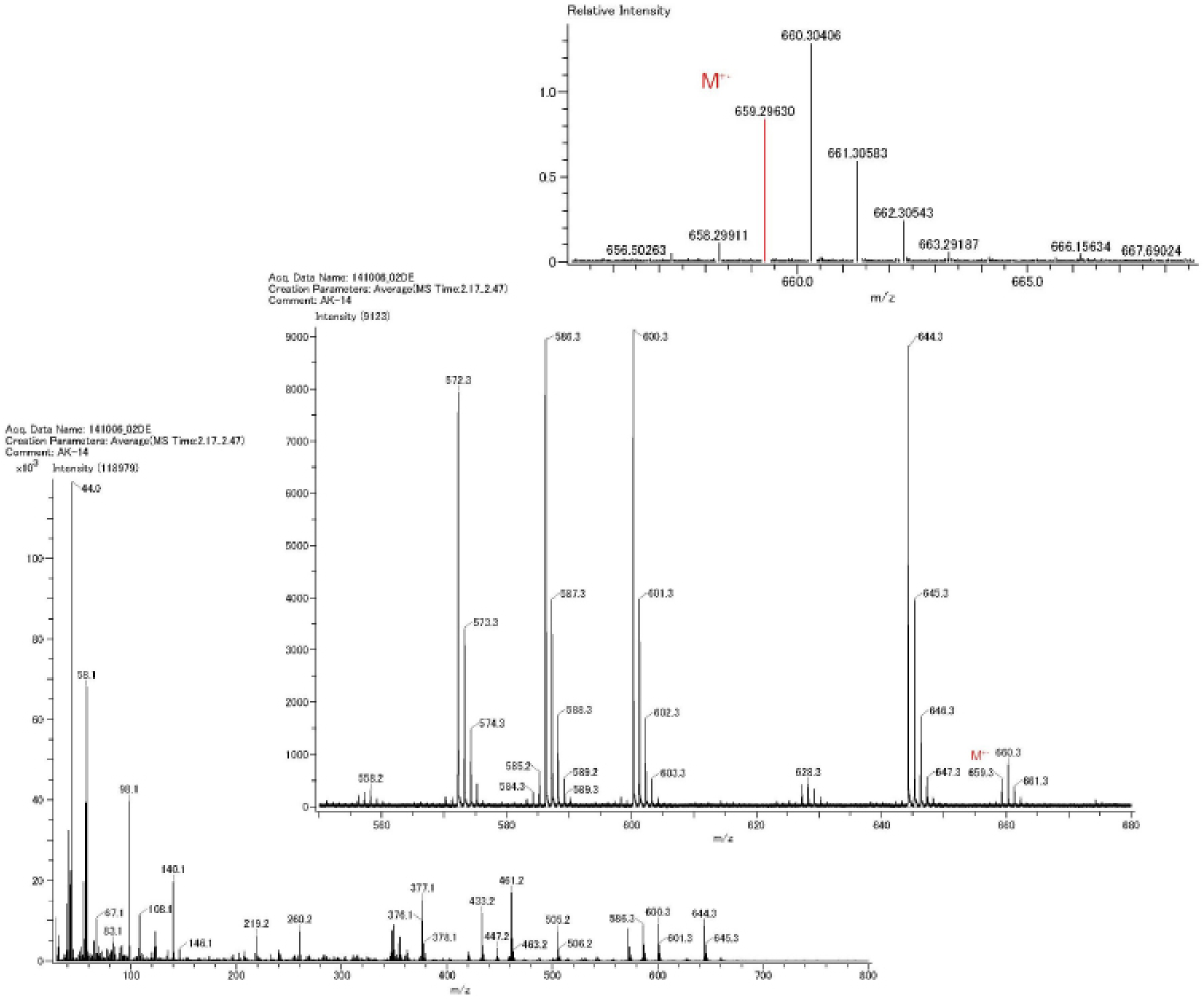
Relative Intensity
M+·
660.30406
659.29630
661.30583
662.30543
663.29187
658.29911
656.50263
666.15634
667.69024
m/z
Acq. Data Name: 141006_02DE
Creation Parameters: Average(MS Time:2.17..2.47)
Comment: AK-14
Intensity (9123)
m/z
Intensity (118979)

(d)

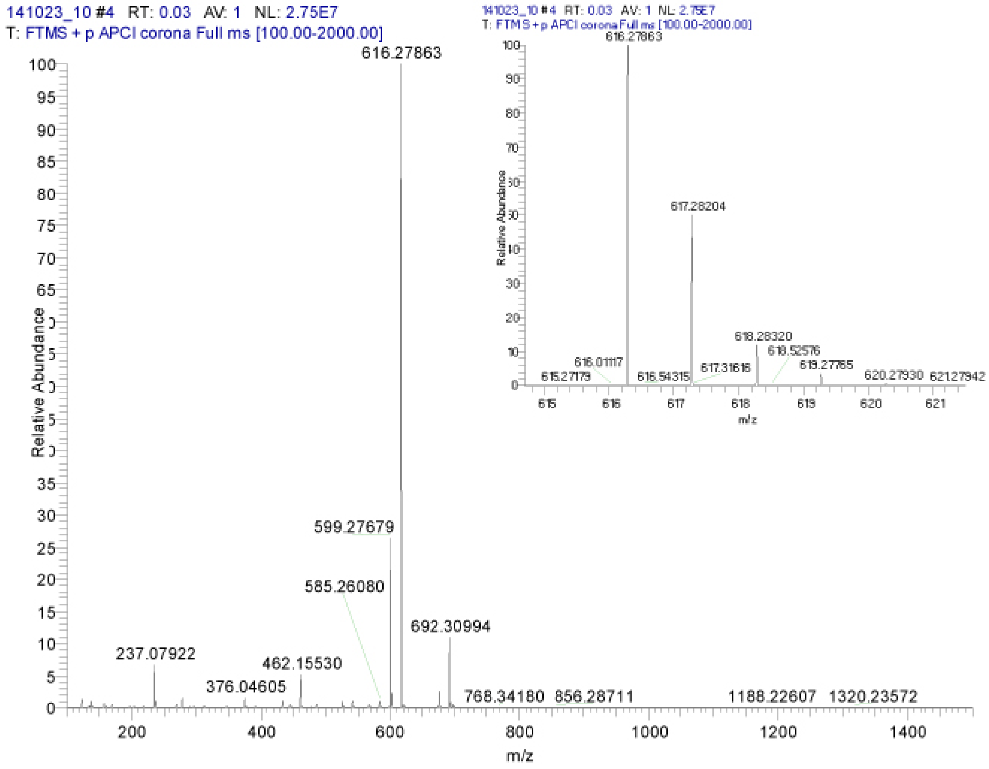
141023_10 #4 RT: 0.03 AV: 1 NL: 2.75E7
T: FTMS + p APCI corona Full ms [100.00-2000.00]
616.27863
599.27679
585.26080
692.30994
237.07922
462.15530
376.04605
768.34180
856.28711
1188.22607
1320.23572
Relative Abundance
m/z
617.28204
618.28320
618.52576
619.27765
620.27930
621.27942
616.01117
615.27179
616.54315
617.31616

(e) **[Positive mode]**

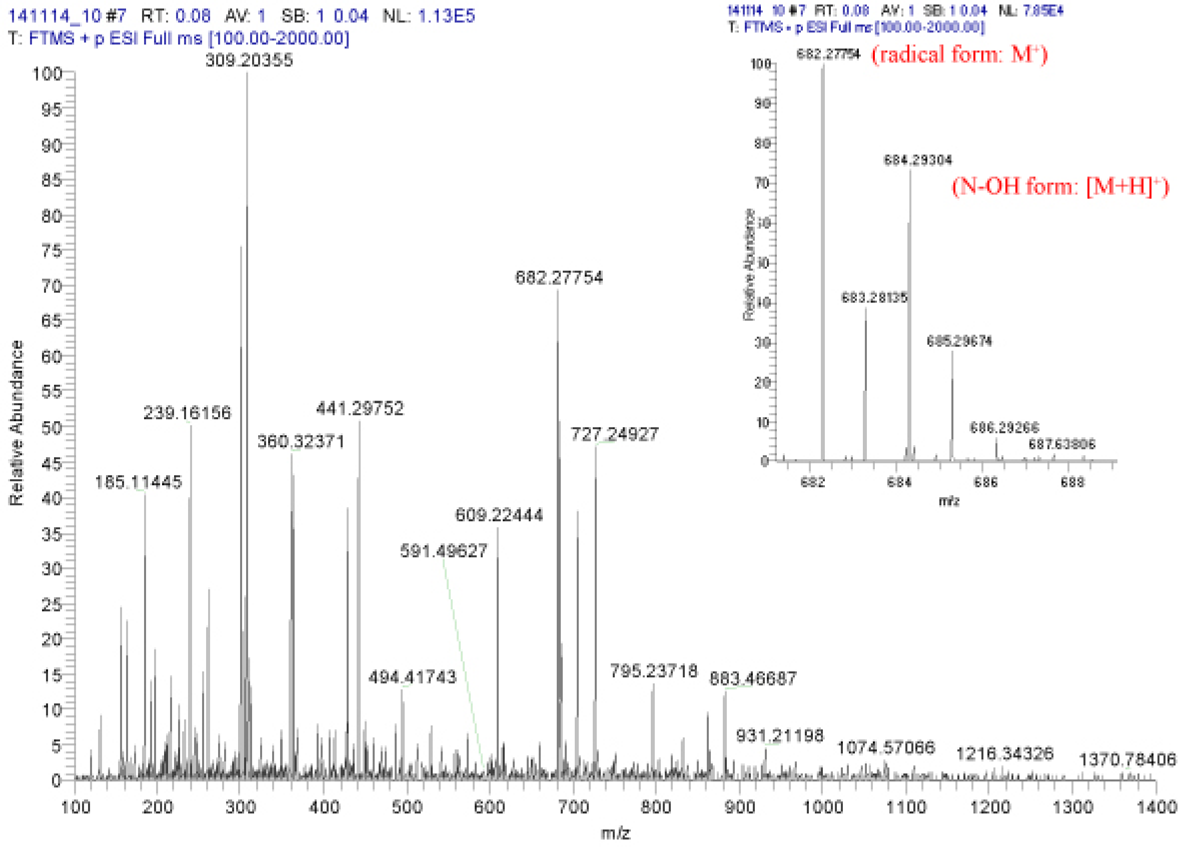

[Negative mode]

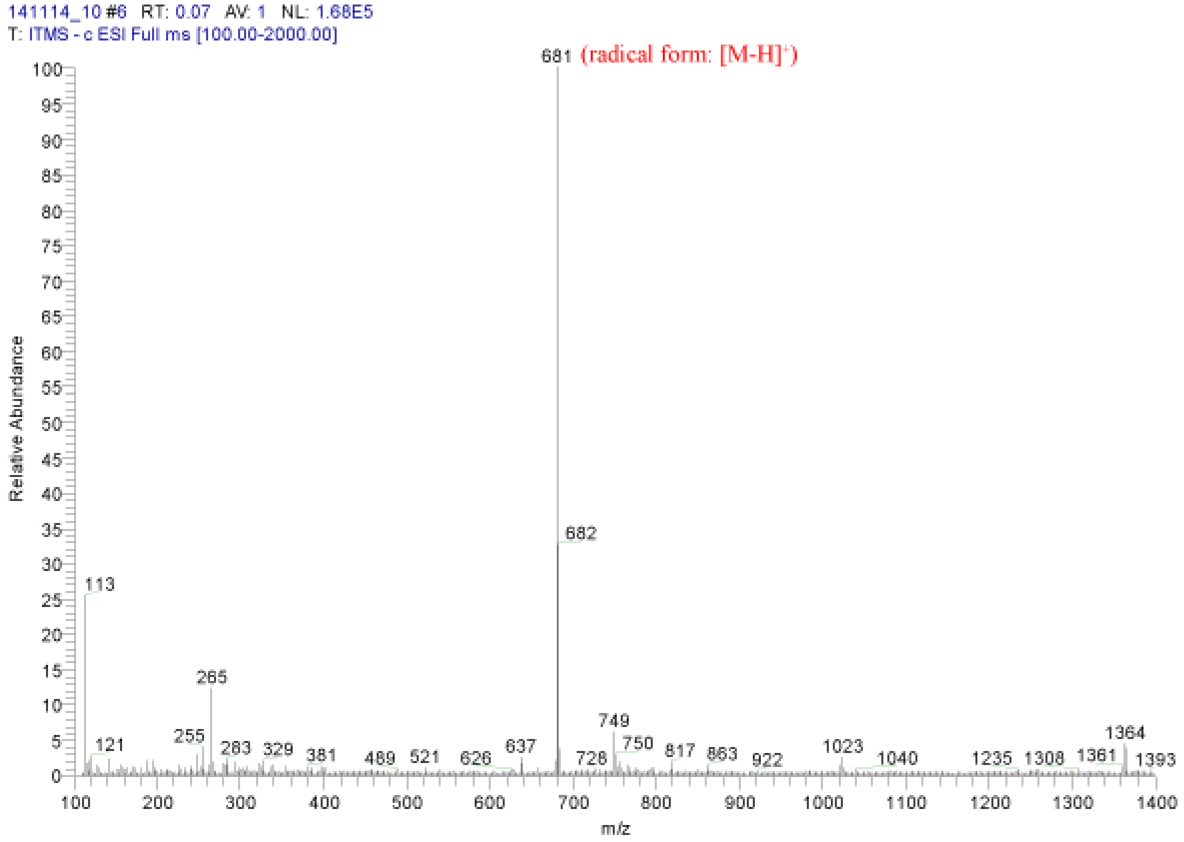

Figure S1. HRMS spectra of (a) compound **2**, (b) compound **4**, (c) compound **6**, (d) compound **7**, and (e) **TEMPO-dye**.
